# The effect of simulation-based training in non-physician anesthetists in Tigray region, Ethiopia

**DOI:** 10.1186/s13104-020-05041-1

**Published:** 2020-04-01

**Authors:** Naod Bulti Etanaa, Kore Menjie Benwu, Hagos Gebregzabiher Gebremedhin, Haftom Berhane Desta

**Affiliations:** Department of Anesthesia, College of Health Sciences, Mekelle University, P.O.Box: 1871, Mekelle, Ethiopia

**Keywords:** Simulation, Non-physician anesthetists, Knowledge, Attitude

## Abstract

**Objective:**

In Ethiopia simulation-based anesthesia education is a new way of teaching method which started in Mekelle University as of January, 2019. Hence, the purpose of this study is to evaluate whether simulation-based training improves non-physician anesthetists’ knowledge and attitude on maternal and neonatal anesthesia cares or not.

**Results:**

Out of 50 study subjects, 66% had a working experience of less than 5 years. Knowledge score improved significantly from 49.78 to 66.22% in pretest and posttest results respectively. The posttest result was significantly improved (P < 0.001) for all knowledge questions. The respondents were asked about a negative statement and a positive statement about the need to have effective closed lope communication, maternal resuscitation and neonatal resuscitation. The attitude score improved from 72.45 to 79.11% in pretest and posttest respectively. From the 9 questions the attitudinal mean score for pretest was 6.52 and posttest 7.12. The null hypothesis of equal knowledge and attitude was rejected, *t* (49) = − 5.54, P < 0.001 and *t* (49) = − 2.25, P < 0.03 respectively.

## Introduction

The practice of anesthesia requires both a high level of vigilance and the ability to deal efficiently and quickly with potentially life-threatening situations that may arise during anesthesia. Some complications associated with anesthesia are so rare that most anesthetists will encounter them only occasionally, if at all, during their professional career. Anesthetists are expected to maintain their competence in managing these complications by reading textbooks, journals or attending appropriate lectures or refresher courses. This is passive learning. However, as in most other walks of life, anesthetists best retain knowledge by active rather than passive learning [1].

Simulation can be defined as something that is made to look, feel, or behave like something else especially when applied to research or education [2]. Simulation is used extensively in industries that involve routine but risky activities. Regular use of simulators in anesthesia will not only maintain an anesthetist’s efficiency, but will also help in dealing with those rare events that demand quick and appropriate actions to prevent them from escalating into disasters [1].

Simulation has a long history in health care education. From learning interview and history taking techniques by using patient actors to learning suturing by sewing make-shift lacerations on pig’s hooves, the importance of preparing practitioners in a controlled and supervised environment prior to clinical education has always been at the forefront of clinical Education [3].

Robertson et al. found that simulation and modified team strategies improves nursing students’ and medical students’ communication skills, including an improvement in identification of effective team skill and attitude towards working together as a team [4].

Medical residents trained to place central venous catheters in simulation improve their clinical performance in the intensive care unit [5]. The translation of knowledge, skills, and attitudes from the simulation-based classroom to clinical care is important to show effectiveness or efficacy and drive curricular change [6].

However, investigations into the effectiveness of simulation-based training for dynamic domains involving high stakes and invasive interventions such as in the fields of anesthesiology, surgery, critical care, and emergency medicine are limited [7].

In Ethiopia simulation-based anesthesia education is a new way of teaching method which is only started in Mekelle University as of January, 2019. Hence, the purpose of this study is to evaluate whether simulation-based training improves non-physician anesthetists’ knowledge and attitude on maternal and neonatal anesthesia cares or not.

## Main text

### Method

The study was conducted in Mekelle University, college of health sciences which is located in Tigray, Ethiopia. The universities anesthesia simulation center was established on January 2019 and has 3 master educators and 6 clinical educators who were trained to run simulations for different groups. A 3-day course was held by department of anesthesia, college of health sciences, Mekelle university and was provided by simulation team for the non-physician anesthetists from 25 May–10 June, 2019.

Anesthetists currently working in different part of Tigray were invited to participate as study subjects. Written informed consent was obtained from participants before data collection, including consent for participation. Refusal of the participation in the study had no consequence on the workshop curriculum.

### Simulation sessions

Nine case scenarios were developed to be conducted. The scenario content was based on set objectives and for each scenario a simulator performance pretest and posttest were created. The pretest and posttest topics included the anesthetic management and recognition of Laryngospasm, Bronchospasm, Total Spinal, Local anesthetic toxicity, Septic shock, Multiple trauma, Maternal hemorrhage, Feta distress, Neonatal resuscitation, Preeclampsia/eclampsia.

Scenarios occurred over approximately 20 min, followed by 40 min debriefing. All debriefing started with reactions, followed by setting the fact and discussion on problems and solutions. After debriefing was conducted it was followed by short summary of the learning objectives with power point presentation or video demonstration.

Orientation about simulation and rules and regulations was held for all participants before the simulation session starts. During 30 min didactics period, medical simulation, patient simulation and rules and regulation were discussed. Participants then were introduced to the VICTORIA^®^ S2200 Package: S2200.PK simulator mannequin, monitors and anesthesia machine and the mock operating room environment.

### Data collection and analysis

Identical pretest and posttest were given to the trainees. The measures were pilot tested with 20 matched pre and posttest for final year anesthesia students.

Knowledge and attitude questions were examined for evidence change and response variation respectively. One question was dropped from the pilot measure (inappropriate material given the case) and one was reworded which led to the final version of the pretest. This pretest was completed on the first day of the training prior to any didactics or simulation scenario. At the end of the 3rd day workshop after the final scenario, the posttest was completed. All data were anonymous but coded with unique ID numbers to allow for comparing individual changes in scores. All participants of the training were included in this study.

Quantitative statistical analysis was performed using SPSS (version 20). All tests were 2-tailed, with significance set at p = 0.05. Paired t-test was used to determine difference between pretest and posttest self-efficacy for participants. The research question of this study is ‘do simulation-based trainings have an effect on non-physician anesthetists?

### Instruments

The knowledge assessment was a 9 test items tool to measure the knowledge retention after the simulation-based training. Question format was multiple choice questions (MCQ) which were prepared by the subject matter specialists in Mekelle University anesthesia department. For the MCQ, items analysis was conducted with item difficulty index (P) and discrimination coefficient. Item difficulty index, value of (P), between 0.2 and 0.9 were considered acceptable. values of (P) < 0.2 were considered difficult question and > 0.9 were considered easy question. Point biserial correlation coefficient was used to determine the strength of association between each item; values greater than 0.2 were considered acceptable in the present study.

The attitude assessment items consist of 9 test items each scored on a 5-point Likert scale from ‘strongly agree’ to ‘strongly disagree’. All items were critically evaluated by subject matter specialists in anesthesia department.

## Results

Out of 50 study subjects, 66% had a working experience of less than 5 years. All of them had bachelor of science (B.Sc.) degree in anesthesia and all of them had no prior exposure to the simulation center. Paired pretest and posttest results were available for all participants (Table 1).

**Table 1:** Background data of participants for simulation-based training in Tigray region from 25 May–10 June, 2019

Characteristics	Frequency	Percentage
Sex		
Male	35	70
Female	15	30
Years of experience (years)		
< 2	12	24
2–5	33	66
> 5	5	10
Number of prior exposures to simulation		
Nil	50	100
1 time		
2 times		
> 2 times		
Educational level		
Diploma in anesthesia	–	–
B.Sc. in anesthesia	50	100
M.Sc. in anesthesia	–	–

Knowledge score improved significantly from 49.78 to 66.22% in pretest and posttest results respectively. The posttest result was significantly improved (P < 0.001) for all knowledge questions (Table 2).

**Table 2:** Pre and post test result on simulation-based training for anesthetists in Tigray region from 25 May–10 June, 2019

Simulation block	Mean score				
	Pretest	Posttest	P value	T value	Df
Knowledge	4.48	5.96	0.001	5.5	49
Attitude	6.52	7.12	0.029	2.25	49

The respondents were asked with sentences that show negative attitude or positive attitude towards the need for effective closed lope communication, managing maternal crisis and initiation of neonatal resuscitation. The attitude score improved from 72.45 to 79.11% in pretest and posttest respectively with mean score for pretest 6.52 and posttest 7.12 (Table 2).

The null hypothesis of equal knowledge and attitude was rejected, *t* (49) = − 5.54, P < 0.001 and *t* (49) = − 2.25, P < 0.03 respectively. Thus, the post-training mean was statistically significantly higher than the pre-training mean (Table 2).

From knowledge items 72% correctly answered Question 1on the pretest and Questions 1, 2, 4, 5,7 and 8 had acceptable pretest/posttest difference index (PPDI) and were very sensitive to instruction. Questions 3, 6 and 9 had a low PPDI which shows the questions addressed the concept most participants had mastered before. Six of the knowledge questions had a discrimination coefficient greater than 0.35 (Additional file 1: Table S1, Additional file 2: Table S2).

## Discussion

Medical Education in this part of developing world is waking up to the reality that there is need for change from mostly knowledge-based education, to focus on skills that are needed to perform duties as health care professional. This has led to changes in medical education with emphasis shifting from teacher-centered to student-centered and from discipline-based to integrated learning to problem-based learning so that the health care provider is equipped with skills to enable them to solve real-life problems in hospital or community [8].

One of the more difficult challenges facing a novice practitioner is assimilating all of the knowledge gained in education and training and applying it to real life patient care scenarios. It has long been appreciated that hands-on experience lends itself to better application of knowledge; this is the foundational principle behind apprenticeship [9].

In the present study the improvement on the knowledge retention was statistically significant (P < 0.001) which shows the simulation-based training had improved the trainee’s knowledge regarding the selected topics were improved after the intervention. This result is in line with many studies done [10, 11] regarding to simulation-based knowledge retention. This is because during the training the trainees had the opportunity to corelate the theoretical knowledge and practical clinical scenario that they observe during the scenario running and also the debriefing session addresses the learning objectives of the scenario every time. Hence, the most effective interventions were those that provided opportunities for learners to discuss concerns, practice, and receive feedback on their skills. In addition, role-playing or simulation can be a key part [12–14].

In this study we used the multiple-choice questions (MCQ) as a means of assessing the efficacy of simulation session knowledge retention which improved in 16% of the mean knowledge score. In a study done in Father Muller medical college in India, the posttest score was significantly improved (P < 0.007) and the mean knowledge score was improved by 51% [15]. The mean knowledge score in the present study is lower than the Indian because in the later study the MCQ was given immediately after the debriefing session whereas on the present study the MCQ was given after the end of 3 days session which might have impacted in the acute knowledge retention of the participants. Hence, a long period of follow-up and recording of the knowledge retention is required to determine the long-term effect of simulation-based training.

Anesthesia is a risky endeavor which puts patient’s normal physiology at stake in order to have a safe surgery. Health professionals need to have the right attitude to patient safety priority all the time. Change in attitude to patient’s safety are also used to monitor the effectiveness of an intervention and to follow development of safety attitude during medical school [16]. Studies have shown that interventions such as an e-learning course can improve medical students to patient safety [17].

In the present study the simulation-based training improved the anesthetists’ attitude significantly (P < 0.03) which is in line with a study done at a single pharmacy school campus in North Dakota State University which used root cause analysis to evaluate pharmacy students improvement in attitude to reduce medication error and promote patient safety [18]. The improvement in the posttest attitude score could be because the simulation-based sessions encouraged participants to actively engage in the real time patient care and during the debriefing session video analysis on what went well and what could have been improved were discussed. Open discussion on the emotional aspect of participants condition were addressed after the completion of each scenario which helped to make participants come at ease at the end of each session.

## Conclusion

Our data demonstrated significant improvement in knowledge and attitude after a simulation-based anesthesia training for non-physician anesthetists. Hence, simulation-based training for non-physician anesthetists can play a big role in the education and development of anesthetists in Ethiopia as it is well aligned with adult learning. Simulation as a way of teaching and learning in our country needs a cultural change in education that need to be measured in the long term. Future similar studies more purposefully focused on long term knowledge retention and positive attitude should be sought.

## Limitations

The survey used small sample size which might have shown an inflated result. Therefore, a further study with an increased sample size should be conducted. The study was conducted in a single institution, which limits its generalizability. Although all instruments underwent preliminary validity and reliability testing for this study, additional evaluation in a more diverse setting is necessary to further refine the general applicability. In addition, although the post simulation-based training was conducted right after the completion of the whole training, further study is required to actually determine knowledge and attitude effect of simulation in non-physician anesthetists in Tigray region.

## Supplementary information

**Additional file 1: Table S1.** Knowledge items used for assessment and proportion of respondents correct response in Tigray region from 25 May–10 June, 2019.

**Additional file 2: Table S2.** Item difficulty and discrimination coefficient on Knowledge items used for assessment in Tigray region from 25 May–10 June, 2019.

## Data Availability

The datasets used and/or analyzed during the current study are available from the corresponding author on reasonable request.

## Abbreviations

ACSH: Ayder Comprehensive Specialized Hospital
BSC: Bachelor of sciences
MSc: Master of sciences
MCQ: Multiple choice question
PPDI: Pretest/posttest diference index

## References

[1] Chopra V, Gesink BJ, de Jong J, Bovill JG, Spierdijk J, Brand R (1994). Does training on an anaesthesia simulator lead to improvement in performance?. Br J Anaesth.

[2] Udani AD, Kim TE, Howard SK, Mariano ER (2015). Simulation in teaching regional anesthesia: current perspectives. Local Reg Anesth..

[3] Cheng A, Duff J, Grant E, Kissoon N, Grant VJ (2007). Simulation in paediatrics: an educational revolution. Paediatr Child Health..

[4] Robertson B, Kaplan B, Atallah H, Higgins M, Lewitt MJ, Ander DS (2010). The use of simulation and a modified TeamSTEPPS curriculum for medical and nursing student team training. Simul Healthcare..

[5] Barsuk JH, McGaghie WC, Cohen ER, Balachandran JS, Wayne DB (2009). Use of simulation-based mastery learning to improve the quality of central venous catheter placement in a medical intensive care unit. J Hosp Med.

[6] McGaghie WC, Draycott TJ, Dunn WF, Lopez CM, Stefanidis D (2011). Evaluating the impact of simulation on translational patient outcomes. Simul Healthcare.

[7] Bruppacher HR, Alam SK, LeBlanc VR, Latter D, Naik VN, Savoldelli GL (2010). Simulation-based training improves physicians’ performance in patient care in high-stakes clinical setting of cardiac surgery. Anesthesiology.

[8] Chacko TV (2014). Moving toward competency-based education: challenges and the way forward. Archiv Med Health Sci.

[9] Boling B, Hardin-Pierce M (2016). The effect of high-fidelity simulation on knowledge and confidence in critical care training: an integrative review. Nurse Educ Pract.

[10] Solymos O, O’Kelly P, Walshe CM (2015). Pilot study comparing simulation-based and didactic lecture-based critical care teaching for final-year medical students. BMC Anesthesiol..

[11] Gaba DM, Howard SK, Flanagan B, Smith BE, Fish KJ, Botney R (1998). Assessment of clinical performance during simulated crises using both technical and behavioral ratings. Anesthesiology.

[12] Ungar L, Alperin M, Amiel GE, Beharier Z, Reis S (2002). Breaking bad news: structured training for family medicine residents. Patient Educ Couns.

[13] Blum RH, Boulet JR, Cooper JB, Muret-Wagstaff SL (2014). Harvard assessment of anesthesia resident performance research G simulation-based assessment to identify critical gaps in safe anesthesia resident performance. Anesthesiology.

[14] Eid A, Petty M, Hutchins L, Thompson R (2009). “Breaking bad news”: standardized patient intervention improves communication skills for hematology-oncology fellows and advanced practice nurses. J Cancer Educ.

[15] Shailaja S, Hilda SS, Pinto PA, D’Cunha RJ, Mahmood LS, Hegde RB (2019). Evaluation of resident satisfaction and change in knowledge following use of high-fidelity simulation teaching for anaesthesia residents. Indian J Anaesth..

[16] Roh H, Park SJ, Kim T (2015). Patient safety education to change medical students’ attitudes and sense of responsibility. Med Teach.

[17] Wallin C-J, Hedman L, Meurling L, Felländer-Tsai L (2009). A-Team: targets for training, feedback and assessment of all OR members’ teamwork. Safer Surg.

[18] Frenzel JE, Skoy ET, Eukel HN (2018). Use of simulations to improve pharmacy students’ knowledge, skills, and attitudes about medication errors and patient safety. Am J Pharm Educ..

