# Supplementary material for: The effect of simulation-based training in non-physician anesthetists in Tigray region, Ethiopia
Source: BMC Res Notes. 2020 Apr 1;13:197. doi: 10.1186/s13104-020-05041-1 (PMC7110791; doi:10.1186/s13104-020-05041-1)
Supplement: Supplementary file 1 — Additional file 1: Table S1. Knowledge items used for assessment and proportion of respondents correct response in Tigray region from 25 May–10 June, 2019. [file 13104_2020_5041_MOESM1_ESM.docx]

**Table S1:** Knowledge items used for assessment and proportion of respondents correct response in Tigray region from 25 May -10 June, 2019.

| **Question** | **Pretest** | | | | **Posttest** | | | |
| --- | --- | --- | --- | --- | --- | --- | --- | --- |
|  | A | B | C | D | A | B | C | D |
|  | n (%) | n (%) | n (%) | n (%) | n (%) | n (%) | n (%) | n (%) |
| 1.A mother who is 38 weeks gestational age is in OR for C/S and her blood pressure is 180/120, has severe headache, visual disturbance, epigastric pain and creatinine level of 2.1mg/dl. Which anesthetic technique is preferable? | 36  (72%) * | 14  (28%) | 0 | 0 | 46  (92%) * | 4  (8%) | 0 | 0 |
| 2. After spinal anesthesia was administered, the patient complained of difficulty of speech, metallic test, and heart rate was 40 bpm. What could be the most likely differential diagnosis? | 2  (4%) | 38  (76%) | 8  (16%) * | 2  (4%) | 0 | 26  (52%) | 24  (48%) * | 0 |
| 3.If a mother is diagnosed with antepartum bleeding, then when would you consider to transfuse blood to her? | 2  (4%) | 33  (66%) | 13  (26%) * | 2  (4%) | 3  (6%) | 24  (48%) | 18  (36%) * | 5  (10%) |
| 4. While you extubate a patient if you hear a loud inspiratory sound, if the reservoir bag is distending and you are unable to ventilate the patient which of the following is incorrect measure? | 9  (18%) | 16  (32%) | 7  (14%) | 18  (36%) * | 7  (14%) | 3  (6%) | 4  (8%) | 36  (72%) * |
| 5. The definitive management to relief intraoperative bronchospasm is? | 0 | 18  (36%) * | 27  (54%) | 5  (10) | 0 | 7  (14%) * | 43  (86%) | 0 |
| 6. If a patient had road traffic accident which one of the following information should you ask before you start anesthesia? | 4  (8%) | 0 | 0 | 46  (92%) * | 0 | 0 | 0 | 50  (100%) * |
| 7. What is the maximum dose of bupivacaine that can be given for peripheral nerve block? | 6  (12) | 16  (32%) | 17  (34%) * | 11  (22%) | 0 | 17  (34%) | 33  (66%) * | 0 |
| 8. While you are doing a neonatal resuscitation and CPR where do you put your fingers to do external cardiac compression? | 13  (26%) | 31  (62%) * | 6  (12%) | 0 | 2  (4%) | 43  (86%) * | 4  (8%) | 1  (2%) |
| 9. Which one of the following statements is incorrect measure to help the fetus before start of anesthesia for a mother having a distressed fetus? | 3  (6%) | 8  (16%) | 2  (4%) | 37  (74%) * | 1  (2%) | 8  (16%) | 0 | 41  (82%) * |

Note: * denotes correct response
