# Supplementary material for: The effect of simulation-based training in non-physician anesthetists in Tigray region, Ethiopia
Source: BMC Res Notes. 2020 Apr 1;13:197. doi: 10.1186/s13104-020-05041-1 (PMC7110791; doi:10.1186/s13104-020-05041-1)
Supplement: Supplementary file 2 — Additional file 2: Table S2. Item difficulty and discrimination coefficient on Knowledge items used for assessment in Tigray region from 25 May–10 June, 2019. [file 13104_2020_5041_MOESM2_ESM.docx]

**Table S2:** Item difficulty and discrimination coefficient on Knowledge items used for assessment in Tigray region from 25 May -10 June,2019.

| **Question** | **Item difficulty (** | | | **Point biserial correlation coefficient** | | |
| --- | --- | --- | --- | --- | --- | --- |
|  | **Pretest**  **(P - value)** | **Posttest**  **(P - value)** | **PPDI**  **(P - value)** | **Pretest** | **Posttest** | **Average** |
| Question 1 | 0.72 | 0.92 | 0.20 | 0.39* | 0.49* | 0.44* |
| Question 2 | 0.16 | 0.48 | 0.32 | 0.18 | 0.66* | 0.42* |
| Question 3 | 0.26 | 0.36 | 0.10 | 0.28* | 0.42* | 0.35* |
| Question 4 | 0.36 | 0.72 | 0.36 | 0.59* | 0.44* | 0.51* |
| Question 5 | 0.36 | 0.86 | 0.50 | 0.32* | 0.1 | 0.21 |
| Question 6 | 0.92 | 1.00 | 0.08 | 0.23 | 0 | 0 |
| Question 7 | 0.34 | 0.66 | 0.32 | 0.1 | 0.21 | 0.15 |
| Question 8 | 0.62 | 0.86 | 0.24 | 0.41* | 0.46* | 0.43* |
| Questions 9 | 0.74 | 0.82 | 0.08 | 0.34* | 0.77* | 0.55* |

Note: * denotes correlation is significant at the 0.05 level

PPDI = (Posttest item difficulty index – Pretest item difficulty index)

.
